# Effects of renal transplantation on erectile dysfunction: a systematic review and meta-analysis

**DOI:** 10.1038/s41443-021-00419-6

**Published:** 2021-06-08

**Authors:** Irham Arif Rahman, Nur Rasyid, Ponco Birowo, Widi Atmoko

**Affiliations:** Department of Urology, Cipto Mangunkusumo General Hospital, Faculty of Medicine, Universitas Indonesia, Jakarta, Indonesia

**Keywords:** Erectile dysfunction, Urogenital reproductive disorders

## Abstract

Erectile dysfunction (ED) is a major global health burden commonly observed in patients with end-stage renal disease (ESRD). Although renal transplantation improves the problem in some patients, it persists in ≈20–50% of recipients. Studies regarding the effects of kidney transplantation on ED present contradictory findings. We performed a systematic review to summarise the effects of kidney transplantation on ED. A systematic literature search was performed across PubMed, Cochrane, and Scopus databases in April 2020. We included all prospective studies that investigated the pre and posttransplant international index of erectile function (IIEF-5) scores in recipients with ED. Data search in PubMed and Google Scholar produced 1326 articles; eight were systematically reviewed with a total of 448 subjects. Meta-analysis of IIEF-5 scores showed significant improvements between pre and post transplantation. Our findings confirm that renal transplantation improves erectile function. Furthermore, transplantation also increases testosterone level. However, the evidence is limited because of the small number of studies. Further studies are required to investigate the effects of renal transplantation on erectile function.

## Introduction

Erectile dysfunction (ED) is defined as the inability to attain or maintain an erection with sufficient rigidity for vaginal penetration and sexual satisfaction [1]. ED is a major health issue in modern life and is often underdiagnosed and underestimated because of the patient’s embarrassment and physician’s unawareness about its high prevalence and effect on the quality of life [2, 3]. ED is frequently observed in men aged ≥40 years, and its prevalence increases with age; its reported prevalence in those aged 65–80 years was varied between 6–55% [2]. The effects of ED on quality of life range from anxiety to depression with a loss of self-esteem [4]. ED is strongly negatively associated with sexual relationship and quality of life of the patients and their partners [5].

Vascular risk factors of ED include age [2], type II diabetes [6], smoking [7], hypertension [8], and dyslipidemia [9]. Although age is associated with increased likelihood of many of the other risk factors of ED, the assumption that ED is a natural concomitant of the aging process is not justified [1].

In addition to the above mentioned factors, ED is frequently observed in patients with chronic illness such as end-stage renal disease (ESRD) [10]. Other common diseases associated with ED include heart disease [11], diabetes [6], hypertension [8], anxiety [4], depression [4], severe malnutrition [12], vitamin and zinc deficiencies [13, 14], uraemic toxins [15], uraemic neuropathy [15], and somatic factors [16]. Overall, ED has been reported in 50% of patients with ESRD, of whom 75% are on chronic dialysis therapy [17]. Although a functional renal graft improves the problem in some patients, as many as 20–50% of recipients continue to suffer from ED [17]. Several factors, such as anxiety [4], side effects of drugs [18], interference with penile vascularity (principally the vascular anastomosis to the hypogastric artery) or response of cavernosal muscle to neurotransmitters [19], and changes in endocrine milieu [20], are the contributing factors. Neuroendocrine disturbances involving the hypothalamic-pituitary-gonadal axis are not reversed by dialysis, however, they are also improved following renal transplantation [17].

Psychogenic factors may also affect erectile function in ESRD patients because approximately one-quarter of these patients may be depressed at any given time [2–4]. Changes in lifestyle such as dietary restrictions, polypharmacy, and hemodialysis predispose patients to depression [2–4]. Without treatment, ED may severely impair the quality of life of both the patient and his partner [2–4]. Fear of failure, conflicts with partners, feelings of guilt, and depression are frequently reported in these patients [2–4]. Successful management of sexual dysfunction in immunosuppressed renal transplant recipients should result in a quality erection, which is adequate for the mutual satisfaction of the patient and his partner, with minimal risk of infection and without compromising the current or future function of the transplanted kidney [21].

To date, studies regarding the effects of kidney transplantation on ED have presented contradictory findings. In most studies, patients regain potency following renal transplantation; however, some studies have reported minimal effects of transplantation on the status of ED [1, 2]. Verification of the effects of transplantation on regaining erectile function can provide therapeutics option for patients with ED. Additionally, if the positive effects of transplantation on ED are proven, patients may be motivated to undergo renal transplantation to simultaneously cure their renal and erectile problems. Therefore, this systematic review aimed to summarise the effects of kidney transplantation on ED and testosterone level.

## Materials and methods

### Description of condition and intervention

The target population included patients with ED with ESRD, and the intervention was renal transplantation to restore adequate renal function. No additional treatments or placebos were administered in the controls in this study. Expected outcome was improvement of ED as assessed using posttransplant international index of erectile function (IIEF-5) scores [22] and nocturnal penile tumescence (NPT) [23] and serum testosterone level [24]. IIEF-5 scores range from 5 to 25, and ED was classified into five categories based on the scores: severe (5–7), moderate (8–11), mild-to-moderate (12–16), mild (17–21), and no ED (22–25) [22]. Furthermore, Rigiscan is a tool to determine NPT [23]. The role of testosterone is to regulate the mechanism of erection in response to sexual desire [24]. Therefore, evaluation of the changes in testosterone levels pre and post transplantation may reflect the effects of the intervention.

### Database search and screening

A systematic literature search was performed to identify studies on erectile functions assessed using IIEF-5 scores, NPT, and serum testosterone levels in patients who underwent renal transplantation. In April 2020, the search was performed in accordance with preferred reporting items for systematic reviews and meta-analyses criteria [25]. The search was conducted in PubMed, Cochrane, and Scopus databases. The general search in PubMed included (‘Renal transplantation’ or ‘Renal transplant’) and ‘Erectile Dysfunction’ and (‘International index of erectile function’ or ‘IIEF-5’), (‘Renal transplantation’ or ‘Renal transplant’) and ‘Erectile Dysfunction’ or (‘Rigiscan’ or ‘Nocturnal penile tumescence’ or ‘NPT’), and (‘Renal transplantation’ or ‘Renal transplant’) and ‘Erectile Dysfunction’ and (‘testosterone’ or ‘Dihydrotestosterone’ or ‘DHT’). Keywords were modified in accordance to each database without any substantial changes that may affect the outcome of the search. Duplicate articles were removed and screening was done to the remaining articles.

### Study selection

All full-text potentially relevant articles were independently assessed by two authors (WD and PB) using predetermined criteria. Inclusion criteria were original studies that reported pre and post transplant IIEF-5 scores, NPT, and serum testosterone levels in a minimum of five patients aged >18 years. Prospective studies were chosen since they have fewer potential sources of bias and confounding than retrospective studies. Studies in animals or those that included pediatric patients were excluded. Multiorgan transplant studies, cross-sectional studies, case reports, studies without concise qualitative outcomes, and grey literature manuscripts not published in peer review journals or books were also excluded.

The search was conducted independently by the two authors (WD and PB). Disagreements regarding search results were resolved by consensus. Titles were screened initially to exclude irrelevant articles. Relevant titles were screened for abstracts alone, and full articles were reviewed if the abstracts did not show sufficient relevance. There were no time period or language restrictions, and all types of original studies other than case reports were eligible. If multiple articles reporting the same data were found, only the most recent or comprehensive papers were included.

### Data extraction and outcome of interest

Data extraction was performed by two authors (NR and IA), and disagreements were settled by consensus. Articles were separated into the following categories based on outcome measures: IIEF-5 scores, NPT, and serum testosterone levels. Data collected for each study included the following: first author, publication year, study design, study period, sample size, population, follow-up duration, and population. Primary outcomes of interest were pre and posttransplant IIEF-5 scores and NPT to assess the erectile function. In addition, the serum testosterone level was also evaluated. Secondary outcomes, which may or may not be available in all studies, were: dialysis duration, use of sildenafil (phosphodiesterase-5 inhibitor), and quality of life. A narrative synthesis was used to analyze the studies, including a description of the characteristics and main outcomes reported in the articles.

### Assessment of methodologic quality

Quality of the included studies was assessed using Central for Evidence Base Medicine checklist for critical appraisal [26]. It included several points, such as PICO (population, intervention, comparison, outcome), randomisation of the samples, similarities between the baseline characteristics of the groups and how each group was treated, was intention-to-treat analyses performed, the blinding method, were the results presented as odd ratios, absolute risk reduction, relative risk reduction, number needed to treat, and the applicability. Furthermore, risk of bias in each study and all the studies combined was identified in terms of selection, performance, detection, attrition, and reporting biases.

### Statistical analysis

Meta-analysis was performed using Review Manager (RevMan) Version 5.3. Copenhagen: The Nordic Cochrane Centre, The Cochrane Collaboration. Results are presented as mean difference with 95% confidence interval (CI) for continuous variables. Heterogeneity was analyzed using chi squared and I-square (I^2^) tests. Data were analysed using the random-effect model when I^*2*^ > 25%, and fixed-effect model when I^2^ was <25%. Statistical significance was considered for *p* values < 0.05. For studies that provided the minimum and maximum values instead of standard deviations (SD) in the mean difference analysis, estimated SDs were calculated using the formula provided by Walter and Yao [27].

## Results

### Literature search

**Fig. 1:**
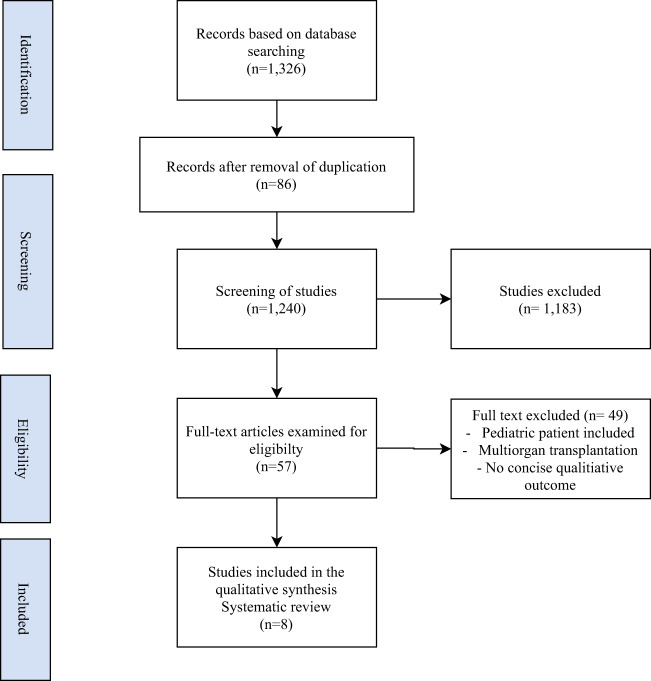
PRISMA flow chart describing the process of identifying pertinent articles PRISMA, preferred reporting items for systematic reviews and meta-analysis.

The initial search produced 1326 papers; of them, 1269 were excluded after removal of duplication and abstract screening, and 57 papers were considered for full-text analysis. Finally, eight papers were included in the systematic review. Details of the electronic search are presented in Fig. 1 while Table 1 showed the quality assessment of the included studies.

**Table 1:** Quality assessment of included studies using CEBM critical appraisal.

Studies	PICO	Randomization	Baseline	Treated	Intention-to-treat	Blind	Results	Applicability
Saha et al. 2002	Yes	No	Yes (treated group only)	Yes	Unclear	Unnecessary	No (only means)	Unclear
Akbari et al. 2003	Yes	No	Yes (treated group only)	Yes	Unclear	Unnecessary	No (only means)	Yes
Shamsa et al. 2005	Yes	No	Yes (treated group only)	Yes	Unclear	Unnecessary	No (only means)	Yes
Mehrsai et al. 2006	Yes	No	Yes	Yes	Unclear	Unnecessary	No (only means)	Yes
Pourmand et al. 2007	Yes	No	Yes	Yes	Unclear	Unnecessary	No (only means)	Yes
Teng et al. 2011	Yes	No	No	Yes	Unclear	Unnecessary	No (only means)	Yes
Eckersten et al. 2017	Yes	No	No	Yes	Unclear	Unnecessary	No (only means)	Yes
Reinhardt et al. 2018	Yes	No	Yes	Yes	Unclear	Unnecessary	No (only means)	Yes

### Study characteristics

**Fig. 2:**
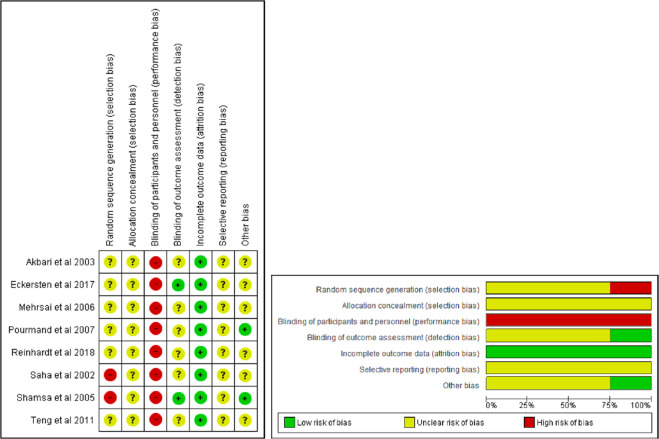
Risk of bias in the included studies.

Overall, 448 patients were included from eight articles. Only one study included the three outcome measures (IIEF-5, NPT, and serum testosterone levels), and four studies included sex hormone levels as their primary outcome. Characteristics of the included studies are summarised in Tables 2, 3, 4. All included studies were published between 2005 and 2018. Risk of bias is illustrated in Fig. 2.

**Table 2:** Systematic review table of selected studies (IIEF score).

Study	Design	Samples	Population characteristics	Patient	Outcome
Pourmand et al. 2007	Prospective cohort	128	Age 42.28 ± 10.4 years (23–63) vs. 42.73 ± 11.2 years (24–64) in the control group. 80/270 men receiving hemodialysis were eligible. Among 80 men there were four deaths (5%), five non function/suboptimal graphs (6.3%), and seven (8.7%) not available for follow-up.	Patients who had been on haemodialysis for at least 6 months with age >20 years and free of comorbidities (DM, history of ischemic heart disease, hypercholesterolemia, history of pelvic trauma or prostate surgery presents of penile deformity, cigarette smoking, uncontrolled major medical illness, previous renal transplantation, and use of medications with significance adverse effects on erectile disfunctions).	IIEF score pre transplantation
					Case: 13.59
					IIEF score post transplantation
					Case: 19.16
					(*p* value of <0.001)
Teng et al. 2011	Prospective cohort	24 of case groups	Mean age 40.8 ± 7.1 years. Mean hemodialysis time priors to renal transplantation 12.83 ± 20.5 months.	Male patients with end stage renal disease awaiting kidney transplants with exclusion of previous or present of DM, peripheral neurological diseases, repeated resting blood pressure >160/95 mmHg, and heart failure functional classification II according to NYHA classification system, continuous peritoneal dialysis priors to kidney transplantation, secondary transplantation, anastomosis of renal graft end-to-end to internal iliac artery, rejection within 3 months after transplantation, primary inability to perform sexually before the onset of renal disease, unavailability during the posttransplant follow-up, unstable postoperative graft function with serum creatinine over 20 mg/L, and sirolimus as part of the immunosuppressive regimen.	IIEF score pre transplantation
					Case: 12.30 ± 9.08
					IIEF score post transplantation
					Case: 22.0 ± 1.00
					(*p* value of 0.046)
Mehrsai et al. 2006	Prospective cohort	128	Mean age for case group 42.3 ± 10.4 years. Mean age for control group 42.7 ± 11.2 years. Mean hemodialysis time prior to renal transplantation 16.8 ± 18.7 months.	Patients who had been on haemodialysis for at least 6 months who underwent kidney transplantation. Age less than 20 years, presence of penile deformities, cigarette smoking, an uncontrolled major medical illness, previous kidney transplantation, type II diabetes, history of ischemic heart disease, hypercholesterolemia, history of pelvic trauma or prostate surgery, and the use of medications that have significant adverse effects on erectile function.	IIEF score pre transplantation
					Case: 13.6 ± 5.2
					IIEF score post transplantation
					Case: 19.2 ± 5.0
					(*p* value of <0.001)
Shamsa et al. 2005	Prospective, interventional, non-randomized study	15	Mean age 35.26 (21–50) years. Mean age for control group not available. Mean hemodialysis time prior to renal transplantation of 4.31 years.	15 consecutive male patients who underwent living donor renal transplants from March 2003 to June 2004 in Mashaad University of Medical Science, Ghaem Hospital, Mashaad, Iran.	IIEF improved in 11 cases (73.33%)
					Was unchanged in two cases (13.33%%)
					Worsen in another two cases (13.33 %)
					(*p* value of not stated)

**Table 3:** Systematic review table of selected studies (Nocturnal Penile Tumescence).

Study	Design	Samples	Population characteristics	Patient	Outcome
Shamsa et al. 2005	Prospective, interventional, non-randomized study	15	Mean age 35.26 (21–50) years. Mean age for control group not available. Mean hemodialysis time prior to renal transplantation of 4.31 years.	15 consecutive male patients who underwent living donor renal transplants from March 2003 to June 2004 in Mashaad University of Medical Science, Ghaem Hospital, Mashaad, Iran.	NPT improved in 11 cases (73.33%) Was unchanged in three cases (20%) Worsen in another one case (6.6%) (*p* value of not stated)

**Table 4:** Systematic review table of selected studies (Testosterone level).

Study	Design	Samples	Population characteristics	Patient	Outcome
Shamsa et al. 2005	Prospective, interventional, non-randomized study	15	Mean age 35.26 (21–50) years. Mean age for control group not available. Mean hemodialysis time prior to renal transplantation of 4.31 years.	15 consecutive male patients who underwent living donor renal transplants from March 2003 to June 2004 in Mashaad University of Medical Science, Ghaem Hospital, Mashaad, Iran.	Pre transplantation hormone level Testosterone: 632.73 ng/d Prolactine: 22.96 ng/ml Post transplantation hormone level: Testosterone: 387.33 ng/d Prolactine: 14.60 ng/ml (*p* value of <0.001 and 0.005 respectively)
Reinhardt et al. 2018	Prospective, non-randomized stuy	97	Mean age of 52.0 (43, 5–61.0) years. Total population was devided based on age groups of ≤50 years and >50 years. Mean duration of dialysis to transplantation of 37.0 (17.5–64.5) months.	42 male patients ≤50 years and 55 male patients >50 years of age underwent living donor renal transplantation from University Hospital Essen, Germany of 1 year research time frame (2017).	Pre transplantation hormone level ≤50 years: 9.5 (6.4–15.4) >50 years: 9.2 (5.8–11.3) Post transplantation, 1 month ≤50 years: 10.2 (8.3–12.2) >50 years: 8.1 (6.3–9.6) Post transplantation, 3 month ≤50 years: 14.4 (11.1–17.4) >50 years: 9.7 (6.9–14.0) Post transplantation, 6 month ≤ 50 years: 13.8 (9.7–16.0) >50 years: 10.4 (8.3–14.5)
Akbari et al. 2003	Prospective, non-randomized study	30	30 men, 24–52 years old. Been on hemodialysis 2–3 times weekly, 4 h each session, >6 months. Duration of hemodialysis before renal transplantation is not stated.	30 men patients undergone kidney transplantation in several hospital (Mirzakoochak Khan Hospital, Sina Hospital, and Imam Khomeini Hospital) in Iran from 1999 to 2001 who were not on erythropoietin usage, non-diabetic, and do not have graft rejection.	Pre transplantation hormone level Testosterone 3.92 ± 2.5 ng/mL LH 8.6 ± 4.6 mIU/mL FSH 9.6 ± 5.1 mIU/mL Prolactin 16.6 ± 10.5 mIU/mL Post transplantation hormone level Testosterone 4.5 ± 1.92 ng/mL LH 7.04 ± 3.32 mIU/mL FSH 8.75 ± 4.76 mlU/mL Prolactin 10.52 ± 5.55 ng/mL (*p* value of 0.005, <0.001, 0.01, <0.001 for all parameters respectively)
Eckersten et al. 2017	Longitudinal, prospective single center, non-randomized study	12	Male patients planned for living donor kidney transplantation between 20–48 years of age (median age of 31 years). Duration of hemodialysis before renal transplantation is not stated.	12 male patients planned for living donor kidney transplantation in Department of Nephrology and Transplantation, Skane University Hospital, Malmo, Sweden.	Pre transplantation hormone level Testosterone 14.9 ± 6.3 nmol/L LH 9.4 ± 4.7 IU/L FSH 3.2 ± 1.5 IU/L Prolactin 516 ± 306 mIE/L Post transplantation hormone level, 3 months Testosterone 17.3 ± 10.7 nmol/L LH 6.6 ± 2.3 IU/L FSH 5.3 ± 3.5 IU/L Prolactin 260 ± 86 mIE/L Post transplantation hormone level, 12 months Testosterone 17.1 ± 7.8 nmol/L LH 6.1 ± 1.7 IU/L FSH 3.9 ± 1.2 IU/L Prolactin 263 ± 85 mIE/L (*p* value of 0.62, <0.05, <0.05, <0.05 for all parameters respectively)
Saha et al. 2002	Prospective study with no clear randomization method stated	14	Male and female patients with age range of 21–60 years. Median age of 39 years. No clear duration of hemodialysis before renal transplantation stated.	14 male patients undergone renal transplantation with measurements of hormone level were done three times: before renal transplantation, at discharged from the transplantation unit, and 6 months after successful transplantation. Median time of discharged of 19 ± 8 days.	Pre transplantation hormone level Testosterone 17.2 ± 5.8 nmol/l LH 14.2 ± 9.4 U/l FSH 6.0 ± 5.7 U/l Prolactin 441 ± 237 mU/l Post transplantation hormone level (immediate measurement) Testosterone 9.6 ± 2.7 nmol/l LH 8.2 ± 3. 3U/l FSH 5.7 ± 3.7 U/l Prolactin 167 ± 71 mU/l (*p* value of <0.01, <0.05, >0.05, <0.0001 for all parameters respectively when compared to pre-Tx level) Pre transplantation hormone level (6 months) Testosterone 17.0 ± 6.0 nmol/l LH 6.4 ± 2.8 U/l FSH 6.9 ± 2.9 U/l Prolactin 216 ± 82 mU/l (*p* value of <0.05, <0.05, >0.05, >0.05 for all parameters respectively when compared to pre-Tx level)

### Improvement in erectile function

**Fig. 3:**
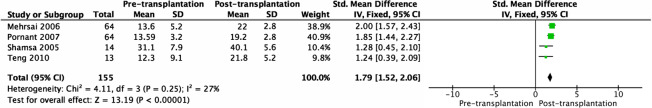
Pre and post transplantation meta-analysis of IIEF-5 scores IIEF-5,International Index of Erectile Function.

IIEF-5 scores in most of the case groups improved post transplantation, although it remained unchanged in two cases and worsened in two cases. Four studies reported mean IIEF-5 values pre and post transplantation. The forest plot in Fig. 3 illustrates that there was a significant difference between pre and post transplantation IIEF-5 values (mean difference: 1.79; 95% CI: 1.52–2.06; *p* < 0.001; Fig. 3). In one study, NPT had improved in 11 patients post transplantation, remained unchanged in three patients, and worsened in one patient.

### Serum testosterone level

**Fig. 4:**
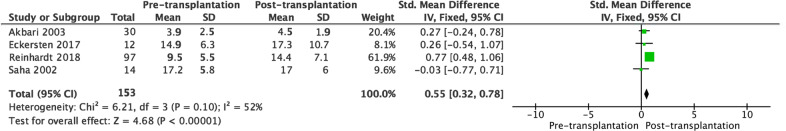
Pre and post transplantation meta-analysis of testosterone levels.

In this meta-analysis, four studies were analysed for differences in the production of testosterone pre and post transplantation. The forest plot in Fig. 4 illustrates that there was a significant difference in serum testosterone levels pre and post transplantation. (mean difference: 0.55; 95% CI: 0.32–0.78; *p* < 0.001).

## Discussion

The effects of renal transplantation on erectile function have been the subject of several studies. Some studies have reported that renal transplantation improves erectile function through the normalisation of endocrine, metabolic, and psychological problems in patients with ESRD who are on heemodialysis. In contrast, others have reported no considerable improvements in erectile function following renal transplantation. This meta-analysis results recommend and strengthen that transplantation in ESRD patients improved erectile function with changes in IIEF scores and NPT as the main benefits found in this study. In addition, significant increase in testosterone level is also found post transplantation.

The present systematic review included eight studies with a total of 230 men who underwent renal transplantation to investigate the effects of renal transplantation on ED. Our results indicate that those who underwent renal transplantation had significantly improved erectile function as reflected in the IIEF-5 scores. The IIEF-5 score is considered by the World Health Organisation to be the most important to address the efficacy of clinical studies because it has high sensitivity and specificity in detecting changes related to treatment in patients with ED [28]. Significant improvements in serum testosterone levels post transplantation were also observed in our review. However, only one study elaborated on the improvements in NPT post transplantation.

Mehrsai et al. [29] conducted a prospective study in 64 patients on hemodialysis and were evaluated before and 6 months after renal transplantation. Erectile function was assessed with the five-item version of IIEF-5. Comparisons were made with a group of age-matched controls before renal transplantation. They found that 56 (87.5%) patients had ED. Successful transplantation improved IIEF-5 scores (13.6 ± 5.2 and 19.2 ± 5.0 before and after transplantation, respectively; *p* value <0.001). The study revealed that severity of ED increased in six (9.4%) patients, eight (12.5%) patients experienced no change in erectile function, and 50 (78.1%) patients reported improved erectile function based on IIEF-5 scores. The study also demonstrated that preoperative IIEF-5 scores and age at transplantation were significantly associated with improvements in ED (*p* value <0.001 and 0.02, respectively).

Another study by Pourmand et al. [30] evaluated the erectile function of 64 patients on hemodialysis between September 2002 and November 2005. Prevalence of ED in the study was 87.5%, and there was no significant association between the duration of dialysis and the severity of ED in the hemodialysis group. Compared with the pretransplant IIEF-5 score, there was significant improvement post transplantation (13.59 vs. 19.16, respectively; *P* < 0.001). These findings were strengthened by the findings of Teng et al. [31] who conducted a prospective cohort study of 24 patients with uremia who underwent kidney transplantation. IIEF-5 score was utilised to assess the erectile function in these patients. They showed that 21 (87.5%) and 11 (45.9%) from varying degrees of ED pre and post transplantation, respectively. Post transplantation IIEF-5 scores were significantly higher than pre transplantation scores (22.00 ± 1.00 and 12.30 ± 9.08, respectively*;* *p* value <0.005) in those who had undergone dialysis for <6 months.

Our results are in line with those of several previous studies that demonstrated improvements in erectile function following renal transplantation. Nassir [32] conducted a study of 52 patients undergoing dialysis therapy and who planned to undergo renal transplantation. There was no improvement in erectile function during dialysis therapy. However, renal transplantation significantly improved erectile function. Shamsa et al. [33] showed that the erectile function of 15 male patients with ESRD improved after successful live donor renal transplant. In a study of 30 patients with ESRD who were on regular hemodialysis and candidates of renal transplantation, Ahmad et al. [34] reported improvements in erectile function after renal transplantation in 40% of patients.

The exact mechanism underlying the effects of renal transplantation on ED remains unclear. However, a previous study [35] found that cavernous artery occlusive disease presented in 78% of patients, and those who underwent arteriography had diffuse atherosclerotic disease of the distal penile arteries. Those who underwent early renal transplantation had vasculogenic impotence only in cases of transplant rejection, which suggests that early transplantation may delay or prevent the development of penile vasculopathy. Beside, it was probably related with the variety of associated risk factors such as: increasing age, diabetes mellitus, peripheral neuropathy, autonomic insufficiency, peripheral vascular disease, pharmacologic therapy, psychologic and physical stresses, anxiety, medication side effects, hypothalamic–pituitary–gonadal axis alterations, anemia, secondary hyperparathyroidism, derangements in arterial supply or venous outflow, and moreover the abnormal structure of cavernous body smooth muscle cells [17].

This systematic review and meta-analysis also emphasized on changes in serum testosterone levels post transplantation in patients on dialysis. It was observed that testosterone levels did not increase in the first 3 months post transplantation; however, an increase was observed at 3 and 6 months post transplantation [33, 36–39]. Transplantation could reverse the uraemic state in patients on long-term dialysis; this was demonstrated by all studies that evaluated serum testosterone levels [36–39]. In contrast, it was shown that serum levels of luteinizing hormone (LH) and prolactin decreased after 3–6 months post transplantation. Furthermore, the effects of renal transplantation on sperm quality and sex hormone levels were analyzed by Akbari et al. [36]. They found that sperm motility significantly improved post transplantation, although morphology and sperm count did not change significantly. Additionally, testosterone levels significantly increased, whereas follicle stimulating hormone (FSH), LH, and prolactin levels significantly decreased after renal transplantation. Results of NPT show improvements post transplantation. Although only one study discussed the correlation between NPT and erectile dysfunction in long term dialysis patient who underwent transplantation [33]. More studies are needed to conclude the correlation between NPT and erectile function. As we only included prospective studies in our review, the evidence is limited because of limited number of studies with a limited number of patients.

Various safe and effective alternative treatments are available for ED in kidney transplant patients. Low-intensity shockwave therapy (Li-SWT) has been proposed to be potential treatment for ED with its capability to promote the formation of new blood vessels and improves cavernosal endothelial function [40]. Hormonal treatment is by far has been reported in improving sexual function in kidney transplant recipients with hypogonadism. Testosterone exerts a crucial double role in male sexual function and promotes the sexual desire by acting at the central nervous system level. In addition, it maintains penile PDE5 expression that triggers the NO cascade, leading to erection [41]. Thus, the possibility of using a combination of testosterone plus PDE5 inhibitors should be considered. However, careful monitoring is required due to the possibility of treatment-emergent adverse events occurrence requiring drug withdrawal [42].

Most of the studies included in the review stated PICO clearly, controlled the baseline characteristics between the groups (clear exclusion and inclusion criteria), and treated each group equally; these points ensure that there was no selection bias. The limitation of this study is the absence of randomization in all the studies, which could be because of the small number of transplantation procedures performed at each center. No intention-to-treat analysis was stated; however, there was no drop out during the follow-up period in each study. Blinding was not necessary because of the objectiveness demonstrated in the results of the studies included in this systematic review and meta-analysis.

In conclusion, our findings confirm that renal transplantation improves erectile function. Significant improvements in IIEF-5 scores post transplantation were proven statistically in this study. However, further larger studies are required to investigate the effects of renal transplantation on ED.
